# MicroRNA Patterns Associated with Clinical Prognostic Parameters and CNS Relapse Prediction in Pediatric Acute Leukemia

**DOI:** 10.1371/journal.pone.0007826

**Published:** 2009-11-13

**Authors:** Hua Zhang, Xue-Qun Luo, Peng Zhang, Li-Bin Huang, Yu-Sheng Zheng, Jun Wu, Hui Zhou, Liang-Hu Qu, Ling Xu, Yue-Qin Chen

**Affiliations:** 1 Key Laboratory of Gene Engineering of the Ministry of Education, State Key Laboratory for Biocontrol, Sun Yat-sen University, Guangzhou, People's Republic of China; 2 The First Affiliated Hospital of Sun Yat-sen University, Guangzhou, People's Republic of China; 3 The Second Affiliated Hospital of Sun Yat-sen University, Guangzhou, People's Republic of China; 4 Institute of Pathology and Southwest Cancer Center, Southwest Hospital, Third Military Medical University, Chongqing, China; 5 Ooparts Corporation, Rockville, Maryland, United States of America; Garvan Institute of Medical Research, Australia

## Abstract

**Background:**

Recent reports have indicated that microRNAs (miRNAs) play a critical role in malignancies, and regulations in the progress of adult leukemia. The role of miRNAs in pediatric leukemia still needs to be established. The purpose of this study was to investigate the aberrantly expressed miRNAs in pediatric acute leukemia and demonstrate miRNA patterns that are pediatric-specific and prognostic parameter-associated.

**Methodology/Principal Findings:**

A total of 111 pediatric bone marrow samples, including 99 patients and 12 normal donors, were enrolled in this study. Of those samples, 36 patients and 7 normal samples were used as a test cohort for the evaluation of miRNA profiling; 63 pediatric patients and 5 normal donors were used as a validation cohort to confirm the miRNA differential expression. Pediatric ALL- and AML-specific microRNA expression patterns were identified in this study. The most highly expressed miRNAs in pediatric ALL were *miR-34a*, *miR-128a*, *miR-128b*, and *miR-146a*, while the highly expressed miRNAs in pediatric AML were *miR-100*, *miR-125b*, *miR-335*, *miR-146a*, and *miR-99a*, which are significantly different from those reported for adult CLL and AML. *miR-125b* and *miR-126* may serve as favorable prognosticators for M3 and M2 patients, respectively. Importantly, we identified a “miRNA cascade” associated with central nervous system (CNS) relapse in ALL. Additionally, miRNA patterns associated with prednisone response, specific risk group, and relapse of ALL were also identified.

**Conclusions/Significance:**

There are existing pediatric-associated and prognostic parameter-associated miRNAs that are independent of cell lineage and could provide therapeutic direction for individual risk-adapted therapy for pediatric leukemia patients.

## Introduction

In recent years, a new class of small noncoding RNAs, ranging from 19 to 25 nucleotides in size and named microRNAs (miRNAs), was discovered and has been shown to regulate gene expression at the transcriptional or post-transcriptional level [1], [2]. The basic function of miRNAs is to reduce the steady-state protein levels of targeted genes. Three primary mechanisms have been proposed based on early observations and experiments involving both siRNAs and miRNAs: translational repression, mRNA cleavage, and transcriptional silencing. The specific consequences of miRNA expression appear to depend on the level of complementarity between the miRNA and the target. Most animal miRNAs contain central regions that are non-complementary to their targets and act by suppressing the translation of the target mRNA [3]–[6].

After the first finding that adult patients with chronic lymphocytic leukemia (CLL) often have deletions or downregulation of two miRNA genes *miR-15* and *miR-16* at 13q14 [7], it has been well known that about 50% of the annotated human miRNAs are located at fragile sites and genomic regions involved in cancers [8], and that miRNA expressions are correlated with various cancers. All these observations suggest that miRNAs function as both tumor suppressors and oncogenes [9], [10]. Additionally, the expression profiling of miRNAs rather than mRNAs has been shown to be a more accurate method of classifying cancer subtypes [10]. Furthermore, cancer-specific miRNA profiles associated with diagnosis, staging, progression, prognosis, and response to treatment were identified in many cancers [11], [12]. In leukemia, microRNA expression signatures associated with the cytogenetics and clinical outcome of adult CLL, AML, and Hodgkin lymphoma were reported [13]–[18].

In comparison with the large number of adult-based studies, the number of studies on the miRNA expression features and functions in pediatric-on set leukemia is relatively low [19], [20]. Mi et al. [21] performed a genome-wide miRNA expression analysis on acute leukemia samples and demonstrated that the expression signatures from as few as two miRNAs could accurately discriminate ALL from AML, and raised the possibility of using such lineage-discriminatory miRNAs to develop a rapid and accurate diagnostic test of ALL versus AML in the future. However, the possibility of a more accurate diagnosis of acute leukemia based on miRNAs in the pediatric population remains to be realized. Similarly, it remains to be determined whether distinct expression signatures exist for prognostic parameters, including prednisone response and specific risk group, and for the association of specific miRNAs with the risk of relapse in pediatric leukemia.

## Materials and Methods

### Patients and Clinical Information

A total of 111 pediatric bone marrow samples, including 99 patients and 12 normal donors, from the First and Second Affiliated Hospital of Sun Yat-sen University were enrolled in this study according to the following criteria: age <14 years at diagnosis; patients with primary ALL or AML; and untreated. Among those samples, 36 patients and 7 normal samples were used as a test cohort for the evaluation of miRNA profiling. The other 63 pediatric patients (31 ALL and 32 AML) and 5 normal donors were used as a validation cohort to confirm the miRNA differential expression. Patients' characteristics, such as presenting age, sex, WBC count, FAB classification, immunological classification, etc., were available for test cohort and validation cohort patients **(see** **Table 1** **,** **Table 2** **)**. Written informed consent for biological studies was obtained from all the patients analyzed. The study was approved by the Ethics Committee of the affiliated hospitals of Sun Yat-sen University.

**Table 1:** Characteristics of test cohort.

Type of sample	Characteristics	Median (range)	No. (%)
**ALL (N = 18)**	**Age at diagnosis, y**	5.4 (1–11)	
	**Sex**		
	Male		14 (77.8)
	Female		4 (22.2)
	**WBC count, ××10^9^/L**	51.6 (3.1–304)	
	Less than 20		11 (61.1)
	20–50		4 (22.2)
	50 or higher		3 (16.7)
	**FAB**		
	L1		7 (38.9)
	L2		8 (44.4)
	L3		3 (16.7)
	**Risk group**		
	HR		4 (22.2)
	IR		10 (55.6)
	SR		4 (22.2)
	**Relapse** &		
	ALL/including CNS relapse		6 (33.4)/4 (22.2)
	Without relapse		12 (66.6)
	**Prednisone response**		
	Good response		12 (66.7)
	Poor response		6 (33.3)
	**Immunophenotype**		
	B		17 (94.4)
	T		1 (5.6)
	**Cytogenetics**		
	BCR/ABL		2 (11.1)
	BCR/ABL negative		16 (88.9)
**AML (N = 18)**	**Age at diagnosis, y**	8.3 (2.5–13)	
	**Sex**		
	Male		9 (50.0)
	Female		9 (50.0)
	**WBC count, ××10^9^/L**	36.7 (3.4–83.8)	
	Less than 10		7 (38.9)
	10–50		6 (33.3)
	50 or higher		5 (27.8)
	**FAB**		
	M1		4 (22.2)
	M2		10 (55.6)
	M3		4 (22.2)
	**Cytogenetics**		
	AML1/ETO		7 (38.9)
	PML/RARA		4 (22.2)
	Both negative		7 (38.9)
**Normal (N = 7)**	**Age at diagnosis, y**	5.8 (1–13)	
	**Sex**		
	Male		4 (57.1)
	Female		3 (42.9)

& With one year of clinical follow-up.

**Table 2:** Characteristics of validation cohort.

Type of sample	Characteristics	Median (range)	No. (%)
**ALL (N = 31)**	**Age at diagnosis, y**	9.5 (0–14)	
	**Sex**		
	Male		25 (80.6)
	Female		6 (19.4)
	**WBC count, ××10^9^/L**	65.7 (1.7–645)	
	Less than 20		20 (64.5)
	20–50		7 (22.6)
	50 or higher		4 (12.9)
	**FAB**		
	L1		15 (48.4)
	L2		14 (45.2)
	L3		2 (6.4)
	**Risk group**		
	HR		14 (45.2)
	IR		9 (29.0)
	SR*		5 (16.1)
	**Relapse^&^**		
	ALL/including CNS relapse		3 (9.7)/3 (9.7)
	Without relapse**		26 (83.9)
	**Prednisone response**		
	Good response**^#^**		17 (54.8)
	Poor response		7 (22.6)
	**Immunophenotype**		
	B		31 (100)
	T		0 (0)
	**Cytogenetics**		
	BCR/ABL		4 (12.9)
	BCR/ABL negative		27 (87.1)
**AML (N = 32)**	**Age at diagnosis, y**	3.7 (0–14)	
	**Sex**		
	Male		23 (71.9)
	Female		9 (28.1)
	**WBC count, ××10^9^/L**	28.9 (2.5–292)	
	Less than 10		18 (56.3)
	10–50		8 (25.0)
	50 or higher		6 (18.7)
	**FAB**		
	M1		5 (15.6)
	M2		13 (40.6)
	M3		8 (25.0)
	M4		3 (9.4)
	M5		3 (9.4)
	**Cytogenetics**		
	AML1/ETO		5 (15.6)
	PML/RARA		5 (15.6)
	Both negative		22 (68.8)
**Normal (N = 5)**	**Age at diagnosis, y**	6.2 (2–11)	
	**Sex**		
	Male		3 (60.0)
	Female		2 (40.0)

* There were no enough total RNA left for three SR patients because they were used for other miRNAs' validation; &With one year of clinical follow-up; ** There were no enough total RNA left for two patients without relapse because they were used for other miRNAs' validation; ^#^There were no enough total RNA left for seven good response patients because they were used for other miRNAs' validation.

### RNA Extraction and MicroRNA Microarray Experiments

Total RNA was isolated with Trizol (Invitrogen, Carlsbad, CA) according to the manufacturer's instructions. The miRNA microarray analyseswere performed with miRNA microarray chips (CapitalBio, Beijing, China) containing 743 probes in triplicate, which corresponded to 576 human (including 122 predicted miRNAs sequences from a published reference^22^), 238 rat, and 358 mouse mature miRNAs found in the miRNA Registry (http://microrna.sanger.ac.uk/sequences/; miRBase8.2; July 2006). Briefly, 4 µg of low-molecular weight RNA was labeled with 5′-phosphate-cytidyl-uridyl-cy3-3′ (Dharmacon, Lafayette, CO) using T4 RNA ligase (New England Biolabs, Ipswich, MA). Hybridization was carried out at 42°C overnight, followed by two consecutive washes with solutions of 0.2% SDS, 2×SSC at 42°C for 5 min and 0.2% SSC for 5 min at room temperature. Arrays were scanned with a LuxScan^TM^ 10K-A laser confocal scanner (CapitalBio) and the obtained images were then analyzed using LuxScan 3.0^TM^ software (CapitalBio). All the data are MIAME compliant and the raw data are being deposited in a MIAME compliant database (ArrayExpress, GEO).

### Date Analysis

Raw data were normalized using the median center tool for genes in the Cluster 3.0 software. Differentially expressed miRNAs were identified by the Significance Analysis of Microarrays (SAM, available at http://www-stat.stanford.edu/~tibs/SAM/ index.html). Array data were clustered using Cluster 3.0. Java TreeView 1.0 was used for tree visualization. Elimination criteria were constructed to detect changes between the leukemia-induced and control miRNA levels. First, the average signal-to-noise ratio (SNR) was greater than two. Second, the adjusted ratios were greater than 2 or less than −2 for inclusion. Third, statistical significance was obtained from the SAM analysis with q<0.01.

### Statistical Analysis

Fisher's exact test, t-test, and chi-square were used to compare baseline characteristics and average miRNA expression between groups of patients, and between patients and controls. All reported P values were two-sided and obtained using the SPSS software.

### Quantitative Real-Time Polymerase Chain Reaction

Quantitative real-time reverse transcriptase PCR (qRT-PCR) was performed as previously described [23] and employed a Hairpin-it^TM^ miRNA Real-Time PCR Quantitation Kit containing a stem-loop-like RT primer, miRNA specific PCR primer, and the Molecular Beacon probe (GenePharma, Shanghai, China). Briefly, RNA was reverse-transcribed to cDNA with an miRNA-specific stem-loop-like RT primer, and the expression of each miRNA relative to the normal control was determined using the 2^−ΔΔCT^ method [24]. Comparative real-time PCR was performed in triplicate and included no-template controls.

### Target Genes Prediction

The target genes prediction was performed to meet both of the following qualifications. First, the miRNA targets were analyzed by using three algorithms, including TARGETSCAN (http://www.targetscan.org/), PICTAR (http://pictar.bio.nyu.edu/), and miRBase (available at http://microrna.sanger.ac.uk/sequences/index.shtml). Second, to reduce the number of false positives, only putative target genes predicted by at least two of the programs were accepted.

## Results

### MicroRNA Expression Profiles in Pediatric ALL and AML

**Figure 1:**
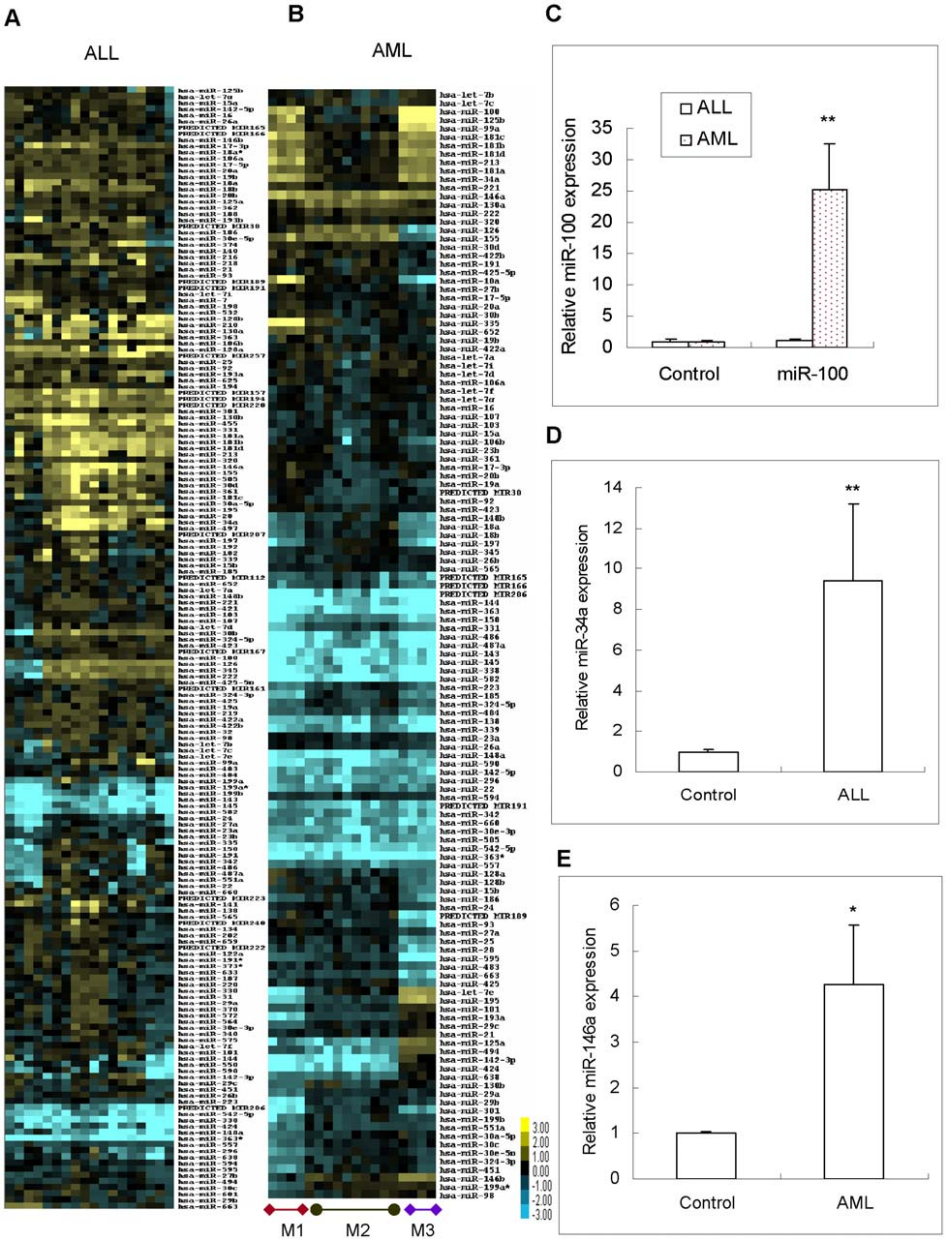
Cluster analysis in pediatric ALL and AML and miRNA expression in pediatric ALL and AML validated with qRT-PCR using the validation cohort. (A) The 182 top–ranked differentially expressed miRNAs in pediatric ALL; (B) The 131 top–ranked differentially expressed miRNAs in the French-American-British (FAB) classification of pediatric AML; (C) MiR-100 expression in pediatric ALL and AML validation cohort (p = 0.000); (D) MiR-34a was up-regulated in pediatric ALL validation cohort (p = 0.003); (E) MiR-146a was up-regulated in AML validation cohort (p = 0.046).

To investigate the expression profile of miRNA in pediatric AML and ALL, we used a miRNA microarray to analyze the miRNome expression in 36 newly diagnosed acute leukemia samples and compared them with the mononuclear cells (MNC) from the bone marrow of seven normal donors **(** **Figure 1A and 1B** **)**. We identified 21 up-regulated and 11 down-regulated miRNAs that showed significant differences between the primary ALL and normal samples, while 17 up-regulated and 18 down-regulated miRNAs were significantly deregulated in primary AML **(** **Table 3** **)** with criteria elimination. To validate these results, we performed qRT-PCR for miR-100 (ALL = 31 and AML = 32, **Figure 1C**), miR-34a (ALL = 24, **Figure 1D**) and miR-146a (AML = 32, **Figure 1E**) using the validation cohort.

**Table 3:** Differentially expressed microRNAs in pediatric AML and ALL compared with the normal control.

MicroRNA	Fold change compared with the normal control*	
	AML	ALL
*miR-100*	24.41	0.75
*miR-125b*	12.37	1.16
*miR-335*	5.35	0.88
*miR-146a*	4.57	4.98
*miR-99a*	3.81	1.79
*miR-34a*	3.21	4.96
*miR-210*	3.12	4.72
*miR-213*	3.12	3.63
*miR-181c*	2.87	2.37
*miR-146b*	2.78	1.91
*miR-126*	2.69	2.09
*miR-181a*	2.55	2.33
*miR-181d*	2.48	3.06
*miR-130a*	2.43	2.17
*miR-195*	2.26	2.95
*miR-181b*	2.09	3.35
*miR-222*	2.00	1.33
*miR-130b*	1.32	3.67
*miR-155*	1.80	3.27
*miR-17-3p*	1.46	2.45
*Let-7a*	1.14	0.59
*miR-15a*	0.73	1.47
*miR-16-1*	0.92	1.25
*miR-199a*	1.37	0.23
*miR-128b*	1.27	3.69
*miR-199b*	1.22	0.22
*miR-128a*	1.17	3.70
*miR-29a*	1.07	0.47
*miR-24*	1.01	0.48
*miR-18b*	0.93	2.23
*miR-28*	0.82	2.34
*miR-424*	0.54	0.31
*miR-331*	0.49	2.41
*miR-505*	0.48	2.45
*miR-150*	0.47	0.99
*miR-30e-3p*	0.46	0.99
*miR-142-3p*	0.46	1.30
*miR-197*	0.45	1.30
*miR-138*	0.40	1.37
*miR-191*	0.38	1.00
*miR-590*	0.36	0.59
*miR-339*	0.36	1.77
*miR-144*	0.33	1.11
*miR-363*	0.33	3.47
*miR-148a*	0.31	0.29
*miR-338*	0.31	0.12
*miR-143*	0.30	0.13
*miR-145*	0.27	0.15
*miR-142-5p*	0.24	0.20
*miR-582*	0.18	0.14

* The expression levels of all miRNAs in the normal control were set as one-fold. High levels are marked in orange and low levels are marked in blue.

The results listed in Table 2 showed that the highly expressed miRNAs in pediatric AML were *miR-100*, *miR-125b*, *miR-335*, *miR-146a*, and *miR-99a*, which were different from those observed in adult AML cases [15], [16], [21]. The most represented miRNAs in the pediatric AML samples were *miR-100* and *miR-125b*, which both had much lower expression levels in ALL. The most highly expressed miRNAs in ALL were *miR-128a*, *miR-128b*, *miR-213*, *miR-210*, *miR-130b*, *miR-146a*, and *miR-34a*. Only 17 miRNAs showed similar expression profiles and were shared by both ALL and AML. These results indicated that the regulatory networks between pediatric ALL and AML could be different.

**Figure 2:**
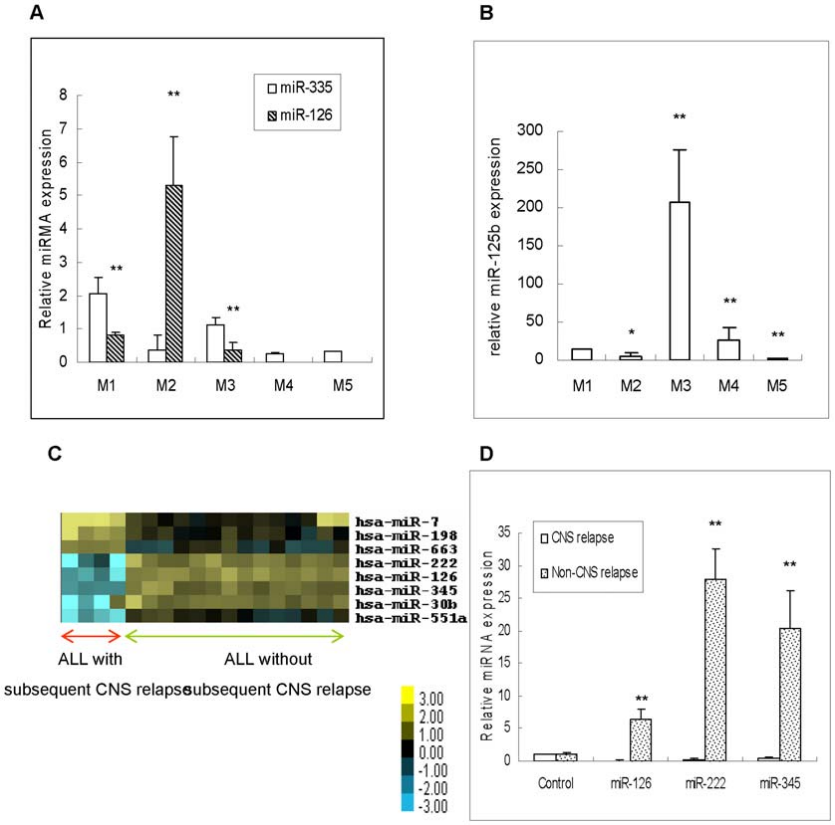
MiRNA expressions in AML subtypes were validated with qRT-PCR using the validation cohort and miRNA expression pattern associated with CNS relapse. (A) MiR-335 and miR-126 expressions in AML subtypes using the validation cohort (the p values for miR-126 were p = 0.006 in M1, p = 0.000 in M2 and p = 0.000 in M3, respectively); (B) MiR-125b expression in AML subtypes using the validation cohort (the p values for miR-125 were p = 0.037 in M2, p = 0.000 in M3, p = 0.000 in M4 and p = 0.002 in M5, respectively); (C) MiRNA expression pattern of pediatric ALL associated with or without CNS relapse; (D) MiR-126 (p = 0.005), miR-222 (p = 0.000) and miR-345 (p = 0.000) were confirmed down-regulated in pediatric ALL patients with CNS relapse compared with non-CNS relapse using the validation cohort.

To explore the relationship among the samples, as well as the underlying patterns of miRNA gene expression, we performed an unsupervised hierarchical cluster analysis using the 576 human mature miRNAs. **Figure 1A** showed the cluster of ALL. The cluster of AML is shown in **Figure 1B**. The unsupervised hierarchical clustering based on the expression of these sets of genes in AML identified three groups that coincided precisely with the phenotypic classification of the most common cytogenetic subtypes of AML lineage, M1, M2, and M3, which are based on the type of cell from which the leukemia developed and its degree of maturity. As shown in **Table 4**, the *miR-335* in M1, the *miR-126* in M2, and the *miR-125b* in M3 were significantly up-regulated. These findings were validated with qRT-PCR using the validation cohort (N = 32) **(** **Figure 2A and 2B** **)**. The differential miRNA expression patterns suggested that the miRNA cascade of FAB subtypes may serve as biomarkers for the classification of pediatric AML. To further investigate the relationship between miRNAs and cytogenetics, we analyzed *miR-125b* in M3 patients with the validation cohort and found that the expression of *miR-125b* was much higher in *PML/RARA* positive patients than in *PML/RARA* negative patients, though *miR-125b* was highly expressed in both groups (**Figure S1A**). This result suggested that *miR-125b* is associated with *PML/RARA* status. The correlation of *miR-126* with cytogenetic *AML1-ETO* in M2 patients was similar to *miR-125b* and *PML/RARA* in M3 patients. **Figure S1B** showed that *miR-126* expression in *AML1-ETO* positive patients was slightly higher than in *AML1-ETO* negative patients using the validation cohort. This implied that *miR-126* may be also associated with the prognosis of M2 patients.

**Table 4:** Differentially expressed microRNAs in the French-American-British (FAB) classification of AML.

MicroRNA	Fold change* in the various AML subtypes		
	M1	M2	M3
*miR-335*	11.74	1.69	1.61
*miR-10a*	7.11	1.37	0.19
*miR-100*	5.05	1.33	47.21
*miR-125b*	2.29	1.09	18.99
*miR-99a*	2.72	1.21	6.73
*miR-155*	2.02	2.23	0.45
*miR-126*	0.88	2.20	0.23
*miR-130*	1.46	2.05	1.04
*miR-146b*	0.57	1.89	2.22
*miR-494*	0.51	0.36	2.01
*miR-125a*	0.30	0.34	2.46
*miR-195*	0.17	0.58	4.31
*miR-7e*	0.13	0.71	3.02
*miR-551a*	0.33	0.61	0.88
*miR-199b*	0.30	0.88	0.83
*miR-29b*	0.13	0.60	0.92
*miR-193a*	0.11	0.36	1.28
*miR-142-3p*	0.01	0.21	1.59

* High levels are marked in orange and low levels are marked in blue.

### MiRNAs May Serve as Biomarkers for Predicting Pediatric ALL Associated with CNS Relapse

We found four cases of ALL with subsequent CNS relapse with one year of clinical follow-up. The miRNA expression pattern was analyzed with SAM. The results indicated that the miRNA expression pattern of ALL with subsequent CNS relapse was significantlyaltered versus non-CNS relapsed ALL **(** **Figure 2C** **)**. There was a significant upregulation of more than 3 fold in *miR-7*, *miR-198* and *miR-633*, while *miR-126*, *miR-345*, *miR-222*, and *miR-551a* were significantly down-regulated in ALL patients with CNS relapse versus non-CNS relapsed ALL. The differential miRNA expression patterns suggested that this miRNA cascade in samples of ALL with subsequent CNS relapse may serve as a biomarker for predicting CNS relapse in pediatric ALL. To confirm the differential expression pattern, we performed qRT-PCR to validate the expression of *miR-126*, *miR-222*, and *miR-345* in the validation cohort samples with or without CNS relapse (N = 3 and N = 26, respectively) as shown in **Figure 2D**. The qRT-PCR data confirmed the microarray results.

### Prediction of Specific Risk Groups in Pediatric ALL by MiRNA Expression Profiling

Pediatric ALL is a heterogeneous disease consisting of various leukemia subtypes that differ markedly in their response to chemotherapy. Accurate assignment of individual patients to specific risk groups will aid in the planning of chemotherapy strategies for patients. To determine whether miRNA expression profiling of leukemic cells could identify known biological ALL-specific risk groups, we analyzed 18 diagnostic bone marrow samples with miRNA microarrays. Remarkably, this analysis clearly identified three leukemia-specific risk groups that were defined as the reference [25]. For standard risk (SR), all of the following criteria must be fulfilled: age >1 year and <6 years at diagnosis; <20,000 leukocytes/µl at diagnosis; the absence of T cell immunology; the absence of t (9,22) or of the BCR-ABL fusion; the absence of t (4,11) or the MLL-AF4 fusion; <1,000 blasts/µl in peripheral blood at D8; and complete remission at D33. For intermediate risk (IR), all of the following criteria must be fulfilled: age <1 year and ≥6 years at diagnosis; ≥20,000 leukocytes/µl at diagnosis; the absence of t (9,22) or the BCR-ABL fusion; the absence of t (4,11) or the MLL-AF4 fusion; <1,000 blasts/µl in peripheral blood at D8; and complete remission at D33. For high risk (HR), all of the following criteria must be fulfilled, in isolation, irrespective of age and initial leukocytes count: blast count >1,000/µl at D8; the absence of complete remission on D33; the presence of (9,22) or of the BCR-ABL fusion; or the presence of t (4,11) or the MLL-AF4 fusion.

**Figure 3:**
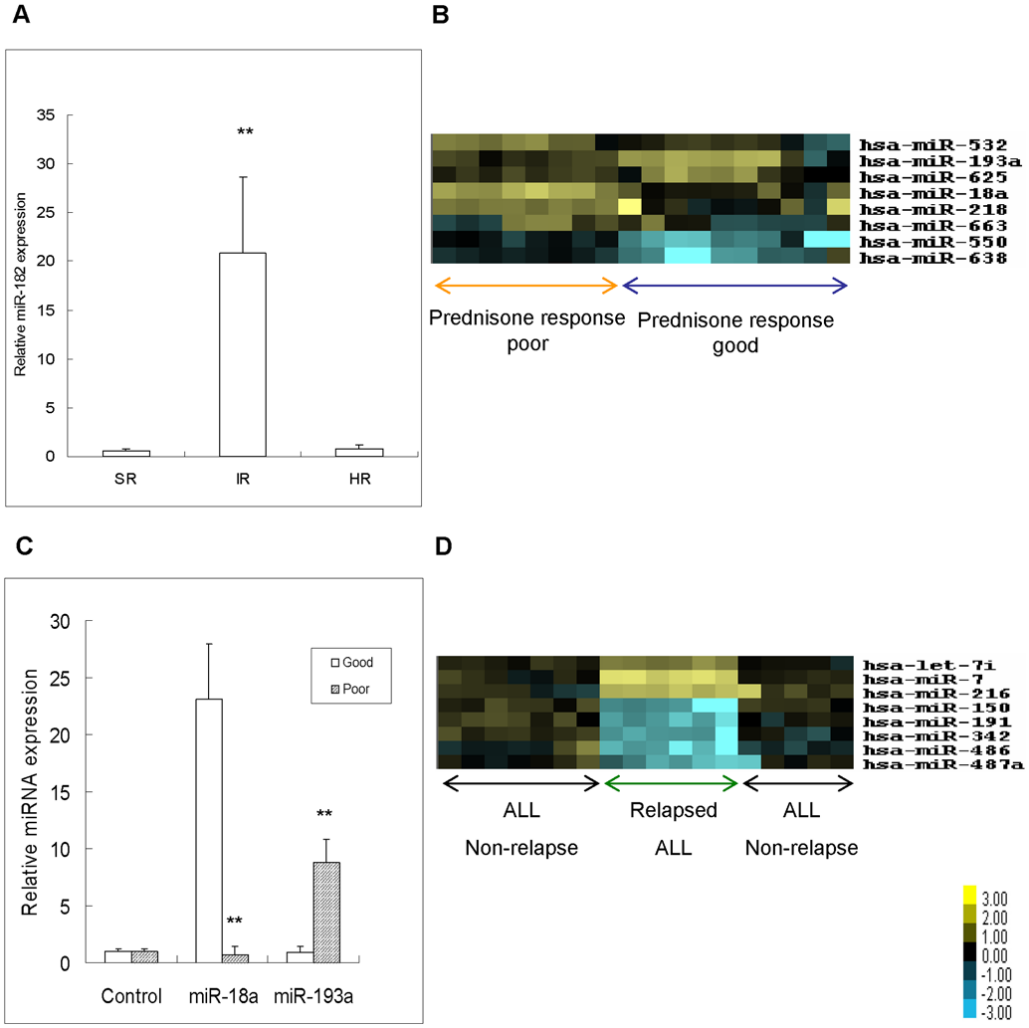
MiRNA expressions associated withz risk group, response to prednisone and relapse in ALL subtypes. (A) MiR-182 was highly expressed in IR groups of ALL (p = 0.000) was confirmed by qRT-PCR using the validation cohort; (B) MiRNA expression pattern of pediatric ALL associated with responses to prednisone treatment Pediatric ALL; (C) Differentially expressed miR-18a (p = 0.000) and miR-193a (p = 0.001) in ALL patients with different responses to prednisone treatment were validated by qRT-PCR using the validation cohort; (D)MiRNA expression pattern in the relapse and non-relapse of pediatric ALL. *p<0.05, **p<0.01.

The greatest number of specific risk group-related miRNA changes was observed in ALL patients. After the first elimination criterion (SNR >2) was applied, only 102 of 576 miRNA remained under consideration. Following the second elimination criterion, the number was reduced to 49, out of which 31 were judged as not statistically significant based on the third elimination criterion. Thus, a total of 18 miRNA species were determined to be abnormally regulated in specific risk groups related to miRNA profiles **(** **Table 5** **)**. For the standard risk group, six abnormally expressed miRNAs were identified, four were expressed at high levels (marked in orange) and two were expressed at low levels (marked in blue), or the intermediate risk group, ten abnormally expressed miRNAs were identified, eight were expressed at high levels and two were expressed at low levels. For the high-risk group, five abnormally expressed miRNAs were identified, three were expressed at high levels and one was expressed at low levels. MiRNA patterns were confirmed by qRT-PCR with selected miRNAs using the validation cohort (**Figure 3A**, N = 28, including SR = 5; IR = 9; HR = 14).

**Table 5:** MicroRNAs associated with risk categories in pediatric ALL.

MicroRNA	Fold change* in various risk factor groups**		
	HR	IR	SR
*miR-210*	7.04	3.78	1.17
*miR-213*	4.53	4.05	0.82
*miR-331*	2.42	3.27	1.92
*miR-182*	0.84	5.29	0.82
*miR-144*	0.69	1.32	3.37
*miR-374*	1.57	1.84	5.06
*miR-361*	1.75	4.72	1.82
*miR-30d*	1.89	4.68	1.82
*miR-141*	1.85	3.62	1.49
*miR-550*	0.33	1.28	0.44
*miR-342*	1.78	1.59	0.38
*miR-125b*	1.23	0.76	0.21
*miR-191*	1.08	2.60	0.24
*miR-27a*	0.72	0.41	0.82
*miR-296*	0.64	0.47	1.63

* High levels are marked in orange and low levels are marked in blue. **SR, standard risk; IR, intermediate risk; HR, high risk.

### The ALL-Related MiRNA Profile is Associated with Prednisone Response

The patient's response to prednisone therapy is the strongest predictor of treatment outcome in pediatric ALL. To verify whether the molecular biomarkers could be used as a reliable stratification factor to predict prednisone response in pediatric ALL, we retrospectively analyzed the outcomes of 18 children with ALL according to the presenting features and initial response to prednisone treatment. The therapeutic effect was defined as a reduction in the leukemic blasts in the peripheral blood to below 1000/µL after 7 days of treatment with prednisone. MiRNA expression data showed that there were significantly different patterns in different prednisone response groups **(** **Figure 3B** **)**. A unique miRNA expression signature composed of 8 genes of the 576 analyzed (*miR-18a*, *miR-532*, *miR-218*, *miR-625*, *miR-193a*, *miR-638*, *miR-550*, and *miR-633*) can differentiate between a good or poor prednisone response in cases of pediatric ALL. We validated the microarray results for selected miRNAs using the ALL validation cohort (N = 24, including Good = 17; Poor = 7) **(** **Figure 3C** **)**. This suggested that miRNA expression can be included among the markers with therapeutic significance in ALL.

### The ALL-specific MiRNA Pattern is Associated with the Prognosis and Disease Progression of Pediatric ALL

Several factors that can predict the clinical course have been defined in CLL [26]–[29]. Calin and Croce described that, in addition to its relevance as a prognostic marker, the miRNA signature may be relevant to the pathogenesis of CLL [30]. The miRNA expression pattern of pediatric ALL was analyzed based on this concept. Although currently available treatments could cure most of the ALL patients in the 1–14 year age group, we found that one third (6/18) of the ALL patients that underwent miRNA profiling relapsed with one year of clinical follow-up **(** **Figure 3D** **)**. The miRNA expression signatures that were identified discriminate between relapse and CR cases with at least a two-fold change in expression. Among them, *miR-7*, *miR-216*, and *miR-100* were significantly up-regulated, while *miR-486*, *miR-191*, *miR-150*, *miR-487*, and *miR-342* were down-regulated in relapsed patients compared with CR cases. Differentiating the cases that relapsed within one year from the others would exactly guide the clinical therapy and effectively improve the cure rate of pediatric acute leukemia. The microRNA cascade of relapse may serve as a biomarker for predicting the relapse of pediatric ALL. To validate this conclusion further studies with a large number of patients are required.

### Putative Target Genes and Their Functions

We selected the best predicted targets for a given miRNA by comparing information from the threebest known target prediction databases: TargetScan, miRBase and PicTar. This approach allowed us to select the common targets found by the three different algorithms. The most significant putative targets were listed in **Table S1**. By examining the Gene Ontology (GO) database, many cancer-associated genes in biological pathways, including apoptosis, cell proliferation, and cell migration were found. For example, *lymphoid enhancer-binding factor 1* (*LEF1*) was found in ALL, *selectin P ligand* (*SELPLG*) was found in AML, and *Bax* was found in prostate cancer.

## Discussion

Recent studies have shown that aberrantly expressed miRNAs are involved in cancer initiation and progression. Their expression profiles can be used for the classification, diagnosis, and prognosis of human malignancies. MiRNA expression profiling using microarray-based methodology has provided new insights into the biology of a variety of adult CLL and AML [14], [15], [16], [21]. Calin and Croce's group revealed that *miR-15a* and *miR-16-1* were differentially expressed between CD5+ and CLL cells. The results also indicated that *miR-15a* and *miR-16-1* were associated with prognostic factors and disease progression, and that these two miRNAs induced apoptosis by targeting *BCL2* [7], [31]. Mi et al. performed a large-scale genome-wide miRNA expression profiling assay and identified 27 miRNAs that are differentially expressed between ALL and AML [21]. However, the possibility of a more accurate diagnosis of acute leukemia based on miRNAs in the pediatric population remains to be realized. In this study, we report the results from the miRNA expression analysis of diagnostic leukemic blasts from pediatric patients with de novo pediatric ALL and AML. Microarray has been proved an effective approch to study the miRNA profiling and helpful to reveal the potential miRNA acting as oncogene or tumor suppressor involved in diseases progress, although there is a high risk of false discovery. However, it is necessary to further validate every abnormal miRNA with a large number cohort. In our study, we provided a global miRNA expression pattern in pediatric leukemia. We did not find abnormal expression of *miR-15a* and *miR-16-1* in either pediatric ALL or AML. The miRNA profiles revealed that ALL and AML had different expression patterns of miRNAs in the pediatric population, although they shared 17 similar expression levels, including six miRNAs with the lowest expression levels. Notably, we found that some miRNAs are differently expressed between pediatric and adult cases, which reveal there exists a pediatric–specific miRNA pattern. For example, *miR-146* has been found down-regulated in adult AML patients [15], while we found that *miR-146a* was up-regulated in pediatric AML patients. Another study showed that miR-335* (not *miR-335*) was down-regulated in 27 adult AML patients [32], however, we found that *miR-335* is up-regulated in pediatric M1 patients. Li et al. found that *miR-100* was up-regulated in adult APL patients, which is the same with pediatric APL patients in our study [33]. However, we also found *miR-100* was up-regulated in pediatric M1 patients, which have not been reported in adult.

So far, the expression of *miR-125b* in adult leukemia is still not very clear. Up-regulated *miR-125b* did not found in adult APL in some studies. Bousquet et al. [34] have shown that myelodysplastic syndrome and acute myeloid leukaemia patients carrying the t(2;11)(p21;q23) translocation are associated with *miR-125b* up-regulation, however, they only focused on 19 adult leukemia patients carrying the t(2;11)(p21;q23) translocation and did not study whether the expressions of *miR-125b* are up-regulated in adult APL patients. Another miRNA expression profiling assay showed *miR-125b* was up-regulated in 7 adult APL patients, but this was not further validated [33]. Thus, the expression of *miR-125b* needs be further examined with large-scale adult APL patients. Our miRNA profiles and qRT-PCR confirmation indicated that *miR-125b* had an abnormally high expression level in pediatric AML, especially in the M3 subtypes. The M3 subtype is characterized by maturation arrest at the promyelocytic stage of development. Analysis of the relationship between *miR-125b* and *PML/RARA* showed that the expression of *miR-125b* was much higher in *PML/RARA* positive patients than in *PML/RARA* negative patients, which suggested that *miR-125b* may serve as a favorable prognosticator in M3. In a very recent paper, Bousquet et al. [34] revealed through in vitro experiments that *miR-125b* was able to interfere with primary human CD34+ cell differentiation, and also inhibited terminal (monocytic and granulocytic) differentiation in leukemic cell lines (HL60 and NB4), demonstrating that *miR-125b* may block the process of differentiation.

Although most ALL patients are cured with the current protocol-based treatments that incorporate systemic therapy, 20–30% of the patients still die after therapy. The poor survival rate of ALL patients is mainly due to early relapses. The management of such patients remains unsatisfactory. Therefore, studies of leukemia molecular pathogenesis in relapsed patients may provide additional insights into their prognosis. We identified a special miRNA expression pattern involved in children experiencing ALL relapse, which included high levels of expression of *miR-7*, *miR-216*, and *miR-100* and low levels of expression of *miR-486*, *miR-191*, *miR-150*, *miR-487*, and *miR-342*. Target prediction indicated that *miR-7* may be associated with a t(1;19)(q23;p13.3) chromosomal rearrangement in pre-B-cell acute lymphoblastic leukemia, and that *miR-216*, *miR-191*, *miR-486*, and *miR-487* putatively caused the suppression of tumor suppressors (**Table S1**). The mechanisms involved in the regulation of these miRNAs have yet to be defined. In addition, we tried to get some clues about the associations between miRNAs involved in prednisone response and overall risk. However, no relevant expression patterns were found between them. A potential reason is possible due to the different definition, especially different time point for patient's responses (for example, for prednisone response, D8 is used for the patient's response to prednisone therapy, but for risk group D33 is used.). As we all know, miRNAs have a high degree of temporal and spatial specificity. So, it is possible that there do exist some miRNAs which are associated with both prednisone response and overall risk but have different expressions in these two stages. This need to be further investigated.

Improved treatment of ALL has virtually eliminated testicular relapse. However, control of the central nervous system (CNS) relapse remains a therapeutic challenge in pediatric ALL [35]. In this study, we intended to elucidate the miRNA signatures involved in the CNS relapse of pediatric ALL. After analysis of the miRNA profiles of ALL patients associated with CNS relapse versus non-CNS relapsed ALL, we identified a cluster of miRNAs with an expression pattern that included high expression levels of *miR-7*, *miR-198*, and *miR-663* and low expression levels of *miR-126*, *miR-222*, *miR-551a*, and *miR-345*. The differential miRNA expression patterns suggested that the miRNA cascade may serve as a biomarker in the detection of early CNS relapse of pediatric ALL. Interestingly, target prediction of the miRNA pattern revealed that some abnormally expressed miRNAs may putatively target neuron function- and neurotransmitter-related genes **(see Table S1**). The remaining questions are what kind of roles are played by the neuron function and neurotransmitter-related genesin CNS leukemia and how are those genes regulated by miRNAs? A clear picture of the regulation–function relationship between these miRNAs and neuron function-related genes needs to be further defined.

In conclusion, we present the results of genome-wide miRNA expression profiling of pediatric ALL and AML from patients with available clinical data. To our knowledge, this is the first study to look at the miRNA expression profiles associated with prognostic parameters in pediatric acute leukemia at the genome-wide level. An obvious difference was identified between some miRNAs' expression of pediatric and adult cases. There exists a pediatric-specific miRNA pattern, suggesting that the spectrum of hematopoietic malignancies between pediatric and adult patients is significantly different. Importantly, we identified a “miRNA cascade” associated with central nervous system (CNS) relapse in ALL. Additionally, miRNA signatures associated with prednisone response, prognosis, and disease progression of pediatric ALL were identified.

## Supporting Information

Table S1 (0.08 MB DOC)

Figure S1 (0.51 MB DOC)
