# Supplementary figures and images for: MicroRNA Patterns Associated with Clinical Prognostic Parameters and CNS Relapse Prediction in Pediatric Acute Leukemia
Source: PLoS One. 2009 Nov 13;4(11):e7826. doi: 10.1371/journal.pone.0007826 (PMC2773830; doi:10.1371/journal.pone.0007826)

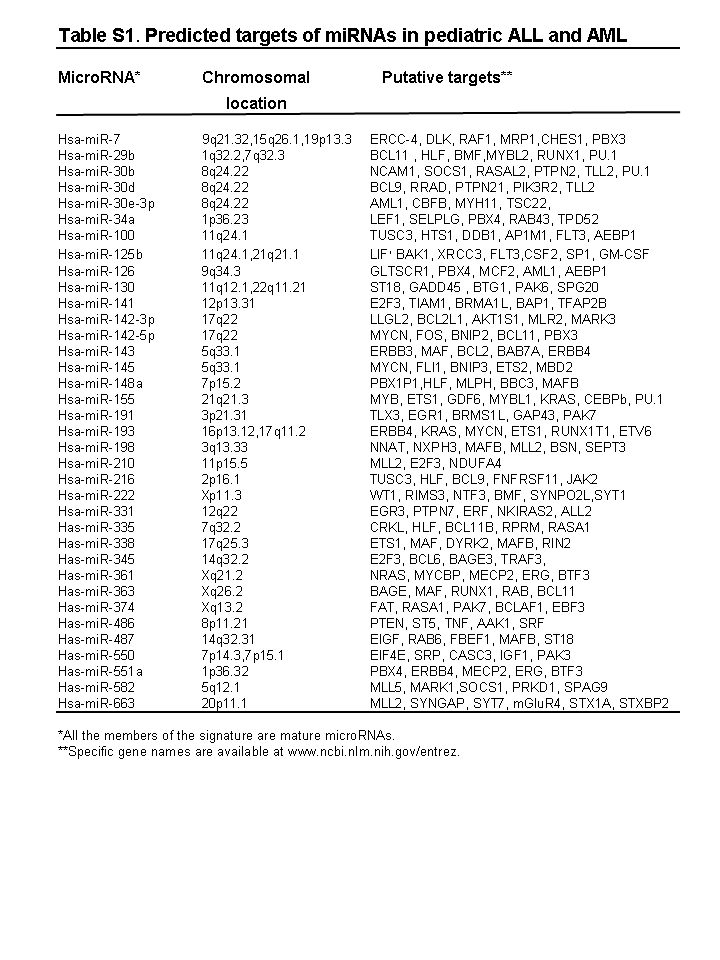

Supplement: Table S1 — (0.08 MB DOC) [file pone.0007826.s001.doc]

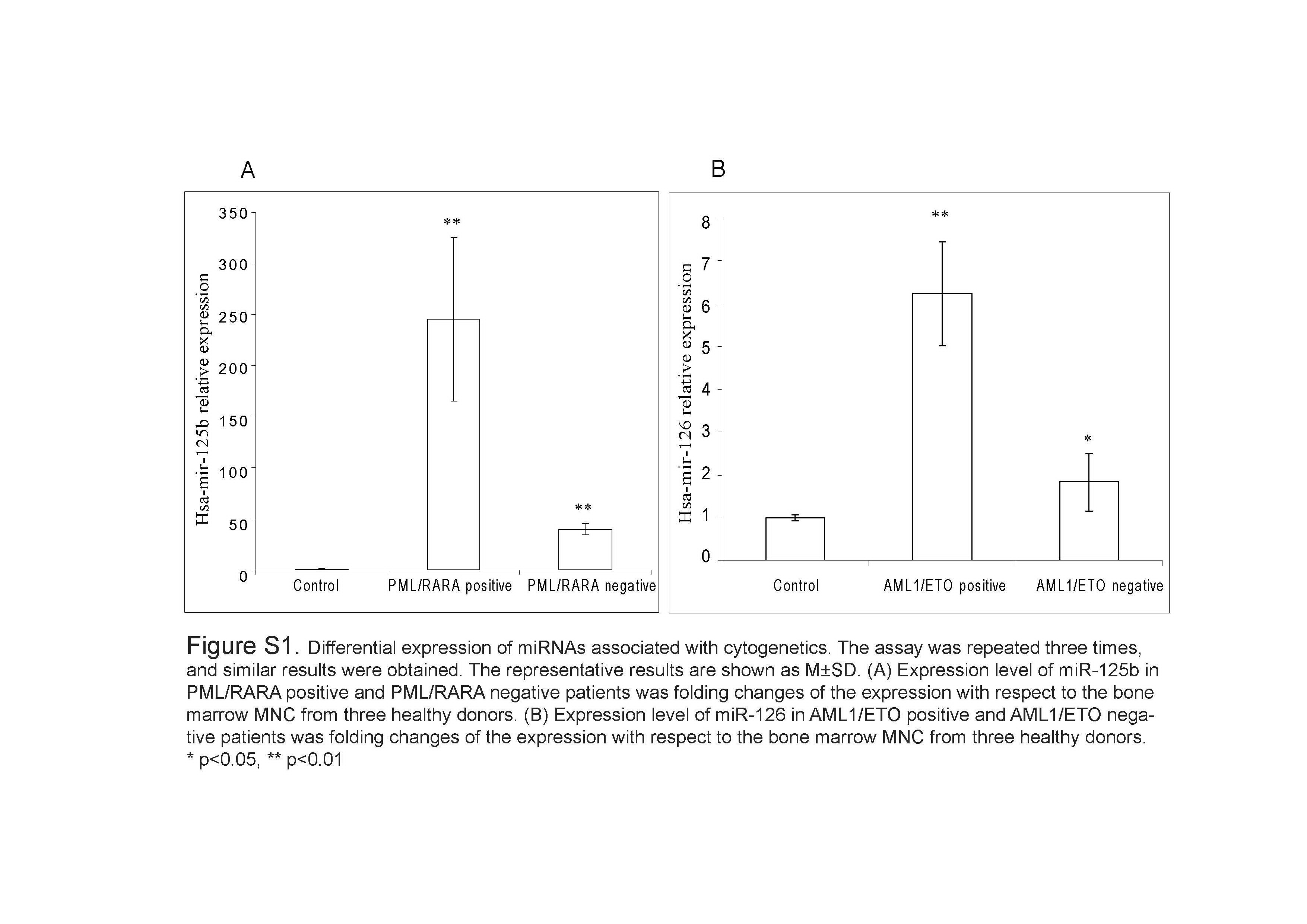

Supplement: Figure S1 — (0.51 MB DOC) [file pone.0007826.s002.doc]
